# COVID-19: An Appeal for an Intersectoral Approach to Tackle With the Emergency

**DOI:** 10.3389/fpubh.2020.00302

**Published:** 2020-06-16

**Authors:** Alessandra Scagliarini, Alberto Alberti

**Affiliations:** ^1^Department of Experimental, Diagnostic and Specialty Medicine, Alma Mater Studiorum, University of Bologna, Bologna, Italy; ^2^Department of Veterinary Medicine, Mediterranean Center for Disease Control (MCDC), University of Sassari, Sassari, Italy

**Keywords:** one health, COVID-19, SARS-CoV-2, disease determinants, public health, vertebrate hosts, epidemics

## Abstract

The knowledge of disease determinants is a pre-requisite for disease prevention. Infectious diseases determinants can be classified in three ways, as: primary or secondary; intrinsic or extrinsic; and associated with host, agent, or environment. In the specific case of COVID-19 several of these determinants are currently unknown leading to difficulties in public health approach to this disease. In this paper, we attempt to address several of the current gaps on COVID-19 using a systematic analysis on recent findings and some preliminary knowledge on animal coronaviruses. A discussion on the impact of COVID-19 determinants in disease prevention and control will be based on the Environmental Change and Infectious Disease (EnVID) systemic framework to address several challenges that may affect the control of the SARS- CoV-2 pandemic spread both in industrialized and in developing Countries.

## Introduction

The ongoing pandemic of a coronavirus-associated acute respiratory disease called coronavirus disease 19 (COVID-19), is the third documented spillover of an animal coronavirus to humans in only two decades (1). In 2011, Weiss and Leibovitz (2) concluded their Chapter on Coronavirus Pathogenesis by asking “Will SARS or another HCoV emerge from its reservoir? It seems like this could happen again given the identification of numerous bat SARS-like viruses and the finding of SARS-like virus in animal such as the civet.” This sentence sounded prophetic, especially now during the COVID-19 pandemic. Globally, COVID-19 is highlighting that preparedness toward pandemics is still not adequate to effectively deal with what is unknown. However, Asian countries that previously experienced similar epidemics (e.g., SARS) demonstrated a prompt and appropriate response aimed at containing viral spread and reducing disease impact.

Indeed, the rapid spreading of the virus across the world has exposed major gaps in the abilities of most countries to respond to a virulent new pathogen. The WHO-China Joint mission report (3) concludes that it is imperative to timely fill the knowledge gaps in the natural history of the disease to put in place effective control strategies (4) such as effective diagnostic tools, vaccines, and antivirals.

SARS-CoV- 2 is (only) the seventh coronavirus known to infect human in spite of the large coronavirus diversity already explored in animals. It is largely established that coronaviruses cause a large variety of diseases in wild and domestic species, both in livestock and companion animals, and in wildlife, leading to significant research in the last half of the Twentieth century (5). Given that SARS-CoV-2 has undoubtedly a zoonotic origin (6), a One Health approach is suggested in order to understand the origin and the causes of the pandemic (7).

This paper aims at analyzing some of the current unknowns on SARS-CoV-2 using recently published data and the available knowledge on coronaviruses infecting animals. Based on current scientific evidence, we elaborate the Environmental Change and Infectious Disease (EnvID) framework analysis (8) to facilitate the identification of relevant environmental and socio-economic factors that may affect disease burden and to provide links to interventions strategies in a One Health perspective.

## The Pathogen

SARS-CoV-2 belongs to the *Coronaviridae* family that comprises enveloped, non-segmented, positive-sense RNA viruses (group 4 in Baltimore classification) named after their corona (crown) like surface, appreciable by electron microscopy, and formed by their largely protruding spike proteins.

According to the International Committee on Taxonomy of Viruses (ICTV) *Coronaviridae* study group (CSG) this family comprises 2 subfamilies (Table S1): *Letovirinae*, including the single species *Microhyla letovirus* 1, infecting the ornate chorus frog *Microhyla fissipes* (9), and *Orthocoronavirinae*, including 4 genera (*Alphacoronavirus, Betacoronavirus, Gammacoronavirus, Deltacoronavirus*) and 38 species. While all *Alphacoronavirus* and *Betacoronavirus* species infect mammals, *Deltacoronavirus* infects exclusively birds. *Gammacoronavirus*, a less diverse genus, includes 2 viral species, the *Beluga whale coronavirus SW1*, and *Avian coronavirus*, infecting, respectively, the beluga whale and birds. To date there is no evidence of coronavirus infection in reptiles, even if the presence of a *Letovirinae* coronavirus in amphibians encourage more coronavirus investigation in reptiles. Coronaviruses infecting human can cause mild respiratory symptoms and conjunctivitis (10, 11), such as *Human coronavirus 229E* and *Human coronavirus NL63* (*Alphacoronavirus*), or mild enteric and respiratory disease, such as *Betacoronavirus 1 OC43* and *Human coronavirus HKU1* (*Betacoronavirus*); whereas *Severe acute respiratory syndrome-related coronavirus* (SARS) and *Middle East respiratory syndrome-related coronavirus* (MERS), both belonging to *Betacoronavirus*, can cause severe disease and are example of spillover of animal viruses to human. COVID-19 associated virus was recognized by CSG as forming a sister clade to the prototype human and bat severe acute respiratory syndrome coronaviruses (SARS-CoVs) of the species *Severe acute respiratory syndrome-related coronavirus*, and it was designated as SARS-CoV-2 (12).

The considerable diversity of coronaviruses is driven by their genetic and evolutionary features. Specifically, mutation and recombination appear to be particularly important in coronavirus evolution (13–16). As RNA viruses, CoVs tend to accumulate random mutations at a far higher rate than their hosts. In spite of the proofreading-repair activity of their polymerase, increasing copying accuracy up to 14-fold (17), coronaviruses form mutant spectra (mutant clouds, quasispecies); viral populations consisting of dynamic and complex mutant distributions, rather than unique genomic sequences (18). Quasispecies correlate to enhanced virulence and evolvability and render difficult to prevent and control viral diseases. The ability to exist as mutant clouds in an individual host have been described both in animal CoVs, such as the bovine enteric and respiratory coronaviruses (19) and in human SARS-CoV (20, 21). Furthermore, during CoV replication template switching favors homologous recombination among different CoVs lineages or with less related viruses. Cooperatively, CoVs circulation in multiple host species may increase recombination events (15). Among others, genetic recombination was demonstrated in animal viruses such as rodents CoVs (13, 22), porcine PEDV (23), cat and dogs CoVs (24, 25), Hedgehog CoVs (26), and bats (16, 27). Notably, recombination between Coronavirus and Orthoreovirus has been postulated in the case of Rousettus bat coronavirus GCCDC1 (28). Genetic recombination in Human CoVs, including, NL63, HKU1, OC43, SARS-CoV, and MERSCoV has also been documented (15, 16). Additionally, the large genome in CoV, and the presence of key mutational and recombination hotspots (15, 26, 29) account for extra plasticity in genome modifications, promoting intraspecies variability, host shifts, and novel CoVs to emerge (14, 15). Evidence for a rapid evolution of SARS-CoV-2 was recently shown by comparing 86 complete or near complete genomes from different parts of the world, depicting the great diversity in viral coding and non-coding regions (30).

## The Hosts

It has been postulated that coronavirus evolution and dissemination is nourished by warm blooded flying vertebrates (bats and birds), ideal hosts for the coronavirus gene source; bats for *Alphacoronavirus* and *Betacoronavirus*, such as SARS-CoV and MERS-CoV, and birds for *Gammacoronavirus* and *Deltacoronavirus* (31, 32). Also, a rodent origin has been proposed for HCoV- OC43 and HKU1 (16).

**Figure 1:**
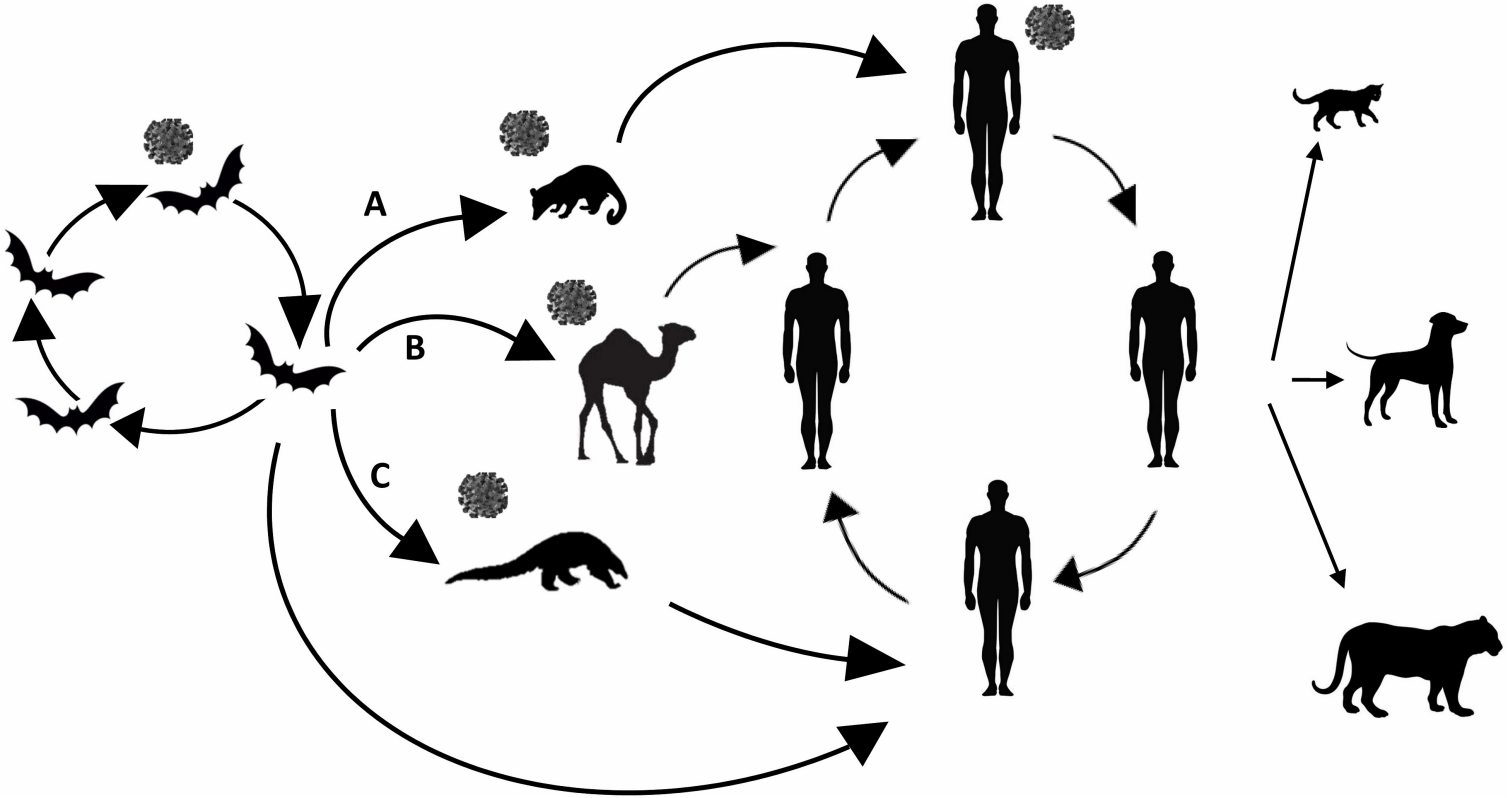
Animal origin of human coronaviruses. SARS- CoV **(A)** emerged from bats, infected civets and humans and adapted to these hosts before causing the SARS epidemic. MERS- CoV **(B)** likely spilled over from bats to dromedary camels. SARS-CoV-2 **(C)** emerged from bats spilled over to human either directly or by previous infecting pangolins. Reverse zoonosis transmission (from human to cats, dogs, and tiger) refers only to SARS-CoV-2.

SARS-CoV emerged in 2002 in southern China (Guangdong Province), as a novel clinical severe disease and rapidly spread to other 28 countries (31, 33). All early cases had a history of contact with living animals (in wet markets or restaurants). Molecular and serological data, and isolation, demonstrated that SARS-CoV originated from civet cats (Figure 1A), family *Viverridae*, in Guangdong market, and also that farmed civets did not play a role as reservoirs (31, 34, 35).

Horseshoe bats family *Rhinophidae* host genetically diverse SARS-like coronaviruses, including ancestors of SARS-CoV and are considered the original source of SARS (31). Moreover, SARS and MERS related CoVs have been identified in *Vespertilionidae* and *Molossidae* bats (36).

Early cases of MERS in Saudi Arabia in 2012; (37) had contact with animals, in particular with dromedary camels (Figure 1B). Molecular and serological data indicated the presence of MERS viruses in dromedaries with high sequence similarities (>99%) to human MERS-CoV (38), and antibodies in camels could be traced back to the eighties (31, 39). More molecular data support that human and camel MERS-CoV isolates belong to the same coronavirus species, and that MERS-CoV originated from the bat gene pool (31). Ancestor analysis suggests that MERS-CoV could have spilled from bats to camels some 30 years ago in Africa, and it was subsequently introduced in the Arabian Peninsula by importing camels from the African continent (40).

Evidence suggests that the SARS-CoV-2 emerged in late 2019 in a wet market in Wuhan, Hubei province, China (41, 42). However, retrospective analyses indicate that SARS-CoV-2 might have spilled somewhere else prior to December 2019 (6). Origin of this virus rapidly became one of the greatest concerns (Figure 1C). The idea of a laboratory-based origin is not plausible as there is no evidence showing that SARS-CoV-2 is a purposefully manipulated virus (11). Also, a snake origin of SARS-CoV-2 can be ruled out as no other coronaviruses have been found in reptiles, and there are not receptor signatures, or other strongly indicative molecular evidence (43) supporting this. It is now recognized that bats such as *Rhinolophus affinis* are natural viral reservoirs, and that the Malayan pangolin (*Manis javanica*) might be the SARS-CoV-2 intermediate host that brought the bat coronavirus to human hosts, even if some studies have proposed that the pangolin, illegally imported into southern China, may be a natural host rather than an intermediate host (44–46). SARS-CoV-2 infection has been demonstrated in two pet dogs in Hong Kong and two pet cats in Hong Kong and in Belgium (47, 48). The pet cases were in close contact with a confirmed COVID-19 human case. Also, a Malayan tiger in Bronx Zoo in New York City developed COVID-19 after exposure to an asymptomatically infected worker (49).

## Transmission Pathways

Person-to-person transmission of SARS-CoV-2 has been documented as for the previously discovered SARS-CoV and MERS-CoV. All these coronaviruses seem predominantly transmitted by respiratory droplets that people cough, sneeze, or exhale over a relatively close distance.

A study conducted by Setti et al. (50) has shown that pollution may have played a role in the propagation of SARS-CoV-2. Researchers evidenced an association between the exceedances of the legal limits of the PM10 concentrations recorded during the 2 weeks preceding the first peak of COVID-19 cases in Northern Italy. This lead could be related to the conditions of airborne particulate matter pollution that may have exerted a boost action on transmission. Animal coronaviruses can replicate in the epithelial cells of both the respiratory and the enteric tracts (51). Enteric tropism was also reported for SARS-CoV-2, causing diarrhea in ~16–73% of patients in addition to respiratory symptoms (52). The transmission of SARS through water droplets from feces via air ventilation systems in Hong Kong was reported (53). Diarrhea and enteric symptoms were also reported in a significant proportion of COVID-19 patients. Recent reports show that SARS-CoV-2 has been detected in stool samples of COVID-19 cases (54–57).

According to WHO and Food Safety Authorities Network (INFOSAN) (58), more information on the potential for persistence of SARS-CoV-2 on foods traded internationally as well as the potential role of food in the transmission of the virus are needed. WHO suggests that the consumption of raw or undercooked animal products should be avoided. Raw meat, raw milk, or raw animal organs should be handled with care to avoid cross- contamination with uncooked foods. Results obtained with SARS-CoV surrogate, Bovine Coronavirus BCoV of the genus Betacoronavirus, showed that contaminated vegetables may serve as a vehicle for transmission through consumption. As an example, Mullis et al. (59) showed that BCoV on lettuce retained infectivity for at least 14 days under household refrigeration conditions. The ability of enteric coronavirus Porcine epidemic diarrhea virus (PEDv) to survive in specific feed ingredients, under modeled conditions simulating shipment, was shown suggesting that contaminated feed ingredients for pigs could serve as transboundary risk factors (60). Extended survival in soybean meal has been confirmed also for swine alphacoronavirus TGEV and deltacoronavirus PDCoV (61). These data lead to speculate that contaminated ready-to-consume produce may be a potential vehicle for zoonotic transmission of coronaviruses to humans. Awareness regarding the possible roles of water, fresh products, and fecal contamination in coronavirus transmission is required at times of human coronavirus outbreaks. The role of animals in SARS-CoV-2 transmission is still debated and need to be clarified. Experimental infection tests conducted in laboratory animals, suggest that SARS-CoV-2 replicates poorly in dogs, pigs, chickens, and ducks, but efficiently in ferrets and cats, with cats transmitting the virus via respiratory droplets (62). For these reasons, a possible role of animal hosts as reservoir and a further source of virus for humans cannot be ruled out, especially in hotspots of biodiversity, and in many developing countries characterized by close proximity among human, wild, and domestic animals.

## Environmental and Socio-Economic Determinants

Urbanization can be considered an important distal environmental factor acting on COVID-19 transmission. According to FAO (63) cities, with their high population density, are vulnerable to the COVID-19 pandemic. The spread of the virus in crowded cities could have extensive morbidity and mortality consequences for urban populations. The very poor and those living in slums have extremely limited access to essential health and sanitation facilities, nutritious food and adequate infrastructure such as piped clean water and electricity. As during the SARS-CoV pandemic (64) SARS-CoV-2 was detected in wastewater collected at a major urban treatment facility in Massachusetts (65). SARS-CoV-2 was detected in sewage of 7 cities and the airport during the emergence of COVID-19 in the Netherlands. Sewage surveillance could be used to monitor the circulation of SARS-CoV-2 complementing current clinical surveillance (66).

Climate is a further distal environmental factor that might indirectly influence SARS-CoV-2 transmission. The WHO assumption is that COVID-19 spread will not ameliorate during summer period. The effect of seasons on transmission of COVID-19 is still unknown. A negative correlation between warmer climate and COVID-19 spread has been suggested by a number of pre-print sources (67–69) and news. However, Caspi and collaborators conclude that their findings, on possible reduced spread during warm season, should be cautiously interpreted and need to be validated as an association between warmer climate and reduced COVID-19 spread might be due to local patterns of transmission rather than by climate.

It has been speculated that the warm months of summer in the northern hemisphere might not necessarily reduce transmission below the value of unity as they do for influenza A given the fact that SARS-CoV-2 R0 was estimated 2–3 (1). SARS-CoV-2 spreads in Countries characterized by tropical climates, such as Singapore. For this reason, winter is not considered a necessary condition for SARS-CoV-2 diffusion and persistence (70). The independence of viral spread from high temperature has been already proved for other coronaviruses such as the SARS- CoV surrogate animal virus transmissible gastroenteritis virus (TGEV) a diarrheal pathogen of swine and mouse hepatitis virus MHV a respiratory and enteric pathogen of mice that showed the ability to survive on surfaces for days at 20°C and wide range of Relative Humidity (RH) levels (20–60%). The animal surrogates showed to be more resistant to inactivation on surfaces than previously studied human coronaviruses, such as 229E. Several studies demonstrated that SARS-CoV and MERS-CoV have the capacity to survive on dry surfaces for a sufficient duration to facilitate onward transmission. SARS-CoV and MERS-CoV were shown to be able to contaminate the environment and fomites, promoting viral access to mucous membranes of the nose, eyes or mouth through individual self-inoculation by hands (71). In fact, SARS-CoV related mathematical and animal models, and intervention studies suggested that contact with contaminated environment is the most important route in some scenarios, such as in health care facilities (72).

SARS-CoV and its surrogates can also survive in environmental reservoirs such as water, foods, and in sewage for extended periods (71, 73–75). SARS-CoV and probably MERS-CoV are shed into the environment at concentrations exceeding the infective dose and they can survive for considerable time on surfaces. The surface survival of SARS/MERS-CoV was shown to be greater than that of other respiratory viruses such as influenza virus. In particular, infective MERS-CoV could still be recovered after 48 h at the 20°C−40% RH condition, whereas the virus remained viable for 8 and 24 h at 30°C−80% RH and 30°C 30% RH, respectively. Instead, H1N1 influenza virus, known to have a seasonal spread pattern, cannot be recovered after 4 h at the same environmental conditions (73).

Economic development should be considered as a further distal factor, FAO (63) stated that there is a particularly high risk of infection for the 1.2 billion people living in the congested and overcrowded informal urban settlements where conditions are already unsafe and unhealthy for human living.

## Discussion

Climate change, urbanization and subsequent loss of natural habitats, changes in human habits and behavior, collectively leading to human and animals living in close proximity, have been identified as the main drivers for the emergence of viral diseases (76).

To some extent COVID-19 outbreak may be considered as an indirect consequence of global environmental changes (77). In fact, multiscale environmental changes encompassing social processes, such as over-crowded urban settings where human and wildlife can come into close contact (e.g., wet markets), trading of exotic animals (6), large-scale population migration, such as those linked to the Chinese New Year celebration (78), were identified as possible causes of the emergence of COVID-19 and of its pandemic spread.

To develop plans and policies for intervention strategies, a preliminary knowledge of disease determinants is necessary (79). COVID-19 was immediately defined as a contact-transmissible infectious disease, spread via direct contact between individuals. Outbreak control measures were thus aimed at reducing the amount of mixing in the population to delay the peak and reduce the final size of the epidemic (80). However, the course of the COVID-19 epidemic is defined by a series of further key factors, some of which are still poorly understood (1). The amount of scientific data produced during the COVID-19 pandemic is amazingly huge, and this can be crucial to translate research into effective public health policies and practices.

According to recent data, COVID-19 is not only linked to person to person transmission, as indirect transmission may also occur. Recent scientific evidence seems to suggest that environmental factors and changes may act as extrinsic determinants in the epidemiology of COVID-19 and of other human and animal coronaviruses.

**Figure 2:**
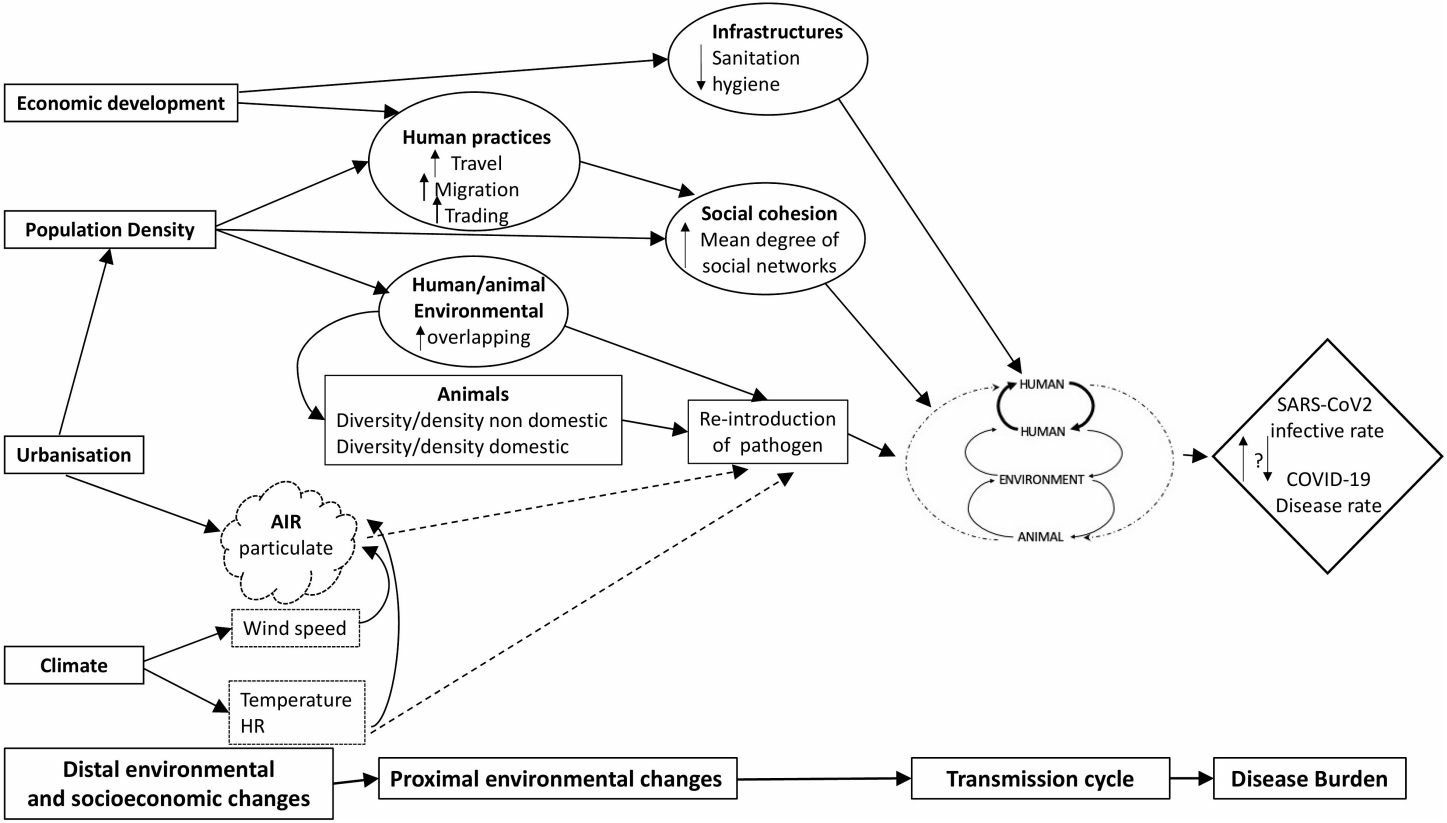
Causal EnvID diagram showing the relationship between distal and proximal environmental changes and COVID-19 disease burden. Solid lines indicate actual influence and scattered lines represent potential influence.

Based on the available scientific literature, we applied the EnvID framework (8) to facilitate the identification of the environment-disease relationships and connections that may impact on disease burden (Figure 2). The EnvID framework encompasses 3 interlocking components including: environment, transmission and disease, and it defines three transmission groups: group I, including directly transmitted diseases; group II, including vector-borne diseases; and group III, including environmentally mediated diseases with non-human host. At first, based on the available data, we attempted to attribute COVID-19 to one of these three groups.

In agreement with the prevalent scientific evidence, COVID-19 might be classified as group I, as it can be transmitted person to person being mainly affected by social processes, such as over-crowding. Social distancing has in fact shown to prevent transmission from symptomatic and non-symptomatic cases, hence flattening the epidemic and delaying the peak.

However, SARS-CoV-2 is also quite resistant in the environment, and the role of non-human hosts as reservoir in infected areas cannot be overlooked. For this reason, it may be also attributed to group III, including those diseases for which transmission can be affected by the modification of human exposure to contaminated environment, media including water, and possibly food and infected animals. Besides indirect contact with contaminated environment, a direct transmission from human to animals and vice-versa cannot be ruled out.

In Figure 2, we represented this transmission dynamic with dashed arrows, as it requires to be better investigated to provide more scientific evidence, in a true One Health approach, where human and veterinary medicine need to collaborate thoroughly to clearly define the whole causal relationships of disease transmission.

According to Brierley et al. (81), transmission routes with environmental components (e.g., fecal–oral or food borne transmission) would be associated with higher virulence than direct, contact-based transmission. EnvID analysis shows that distal environmental changes, such as those related to urbanization and climate, need to be considered as they may act through multiple intermediate steps on COVID-19 transmission. As an example, pollution linked either to urbanization and to climate factors may play a role. It is known in fact that a prolonged exposure to air pollution leads to a chronic inflammatory stimulus, even in young and healthy subjects (82). For this reason, a contribute of air pollution and particulate cannot be neglected as an environmental factor that may directly or indirectly influence the transmission cycle of SARS-CoV-2. Regarding climate, it has been shown that SARS-CoV and MERS-CoV are quite resistant even at a temperature of 30°C when RH is not exceeding 30%. It has been shown for influenza virus that at low RH, evaporation of water from exhaled bioaerosols would occur rapidly, leading to the formation of droplet nuclei; conversely, at high RH, small respiratory droplets would take on water, increase in size and settle more quickly out of the air (83). If SARS-CoV-2 will also show similar resistance patterns, as the other CoVs, and its transmission is not impaired at 30°C, this may suggest that contact-based spread may predominate in the tropics where RH is generally high, whereas aerosol transmission may play a larger role in temperate climates. Even if person to person transmission is considered the main transmission pathway, our proposed transmission pattern underlines that human, environment and animal, might all play a role in the potential spread and persistence of SARS-CoV-2 with a different weight in different geographical and social contexts. In particular, environmental contamination and human/animal habitat overlapping cannot be overlooked especially in developing countries. Coronaviruses exist and can maintain their viability in sewage and wastewater, originating from the fecal discharge of infected patients, highlighting the importance of sanitation to protect public health (84). In developing countries, where water and sanitation systems are often insufficient or ineffective, it is necessary to consider that SARS-CoV-2 transmission might be amplified through water contamination in cities.

Attribution of the burden of disease to environmental risks, highlights in fact the importance of environmental protection for people's health and can inform priority setting for targeted management of environmental determinants (85). The public health measures to contain COVID-19 spreading are being based on the scientific data currently available that mainly derive from studies on the infection's dynamics observed in industrialized countries. An important bias on the large amount of data produced in China and other Asian countries and the lower contributions from Europe and North America is evident. This is clearly due to the timing and geographic spread of the epidemic. As suggested by the EnvID framework, distal and proximal environmental factors, transmission dynamics and consequent morbidity and lethality may differ in different socio-economic contexts. It is thus essential to collect data on the determinants of infection even in developing countries where the capacities of the healthcare infrastructures, diagnostic and research capacities are very limited. In fact, country socio-economic profiles were shown to have an influence on the growth rate of epidemics so that R0 might differ in different geographic areas (70). As an example, FAO warns that the policies to limit the effects of the virus, successfully applied in many industrialized Countries, and based on lockdowns and physical distancing, can spell disaster for the livelihood of individuals and families in developing Countries, leading to food insecurity and deficient nutrition.

Tackling with environmental risk factors always entails intersectoral collaborations and a One Health approach. Research inputs from both human and animal health sides, including many other scientific and non-scientific stakeholders, will be needed to facilitate a systemic way to effectively deal with COVID-19 emergency. A One Health perspective is foreseen, especially in the Global South, to design and implement research programs and policies in which multiple sectors communicate and work together to achieve better public health outcomes.

## Data Availability Statement

Publicly available datasets were analyzed in this study. This data can be found in the EnvID model which is presented as a figure and discussed in the text.

## Author Contributions

AS shaped the conceptualization. AS and AA each contributed to research, critical analysis, and writing of the article. All authors contributed to the article and approved the submitted version.

## Conflict of Interest

The authors declare that the research was conducted in the absence of any commercial or financial relationships that could be construed as a potential conflict of interest.
