# Supplementary material for: COVID-19: An Appeal for an Intersectoral Approach to Tackle With the Emergency
Source: Front Public Health. 2020 Jun 16;8:302. doi: 10.3389/fpubh.2020.00302 (PMC7308477; doi:10.3389/fpubh.2020.00302)
Supplement: Supplementary file 1 [file Table_1.DOCX]

Table 1: Family Coronaviridae: taxonomy and selected epidemiological features

|  |  |  |  |  |  |  |  |
| --- | --- | --- | --- | --- | --- | --- | --- |
| **Subfamily** | **Genus** | **Subgenus** | **Species** | **Host** | **Reservoir/**  **Origin** | **Pathogenicity** | **References^1^** |
|  |  |  |  |  |  |  |  |
| *Letovirinae* | *Alphaletovirus* | *Milecovirus* | *Microhyla letovirus 1 (MLEV-1)* | Ornate chorus frog  *Microhyla fissipes* | Frogs | Not established | Khulud *et al*., 2018 |
|  |  |  |  |  |  |  |  |
| *Orthocoronavirinae* | *Alphacoronavirus* | *Colacovirus* | *Bat coronavirus CDPHE15* | Little brown bat  *Myotis lucifugus* | Bats | Not established | KF430219 |
|  |  | *Decacovirus* | *Bat coronavirus HKU10* | Leschenault's rousette fruit bat  *Rousettus leschenaultii*  Pomona leaf-nosed bats *Hipposideros Pomona* | Bats | Not apparent symptoms but loss of weight | Lau *et al*., 2012 |
|  |  |  | *Rhinolophus ferrumequinum alphacoronavirus HuB-2013* | Greater horseshoe bat  *Rhinolophus ferrumequinum* | Bats | Not established | Wu et al., 2018 |
|  |  | *Duvinacovirus* | *Human coronavirus 229E* | Human | Bats | Common cold symptoms | Hamre and Procknow, 1966 |
|  |  | *Luchacovirus* | *Lucheng Rn rat coronavirus* | Brown rat  *Rattus norvegicus* | Bats | Not established | Wang *et al*., 2015 |
|  |  | *Minacovirus* | *Ferret coronavirus* | *Ferret*  *Mustela putorius* furio | Bats | Epizootic catarrhal enteritis, systemic disease | Williams *et al*., 2000 |
|  |  |  | *Mink coronavirus 1* | American Mink  *Mustela vison* | Bats | Epizootic Catarrhal Gastroenteritis | Vlasova et al., 2011 |
|  |  | *Minunacovirus* | *Miniopterus bat coronavirus 1* | Large Bent-*winged* Bat  *Miniopterus magnater*  Small bent-*winged* bat  *Miniopterus pusillus* | Bats | Not established | Chu *et al*., 2008 |
|  |  |  | *Miniopterus bat coronavirus HKU8* | Large Bent-*winged* Bat  *Miniopterus magnater*  Small bent-*winged* bat  *Miniopterus pusillus* | Bats | Not established | Chu *et al*., 2008 |
|  |  | *Myotacovirus* | *Myotis ricketti alphacoronavirus Sax-2011* | Rickett's big-footed bat  *Myotis ricketti* | Bats | Not established | Wu et al., 2018 |
|  |  | *Nyctacovirus* | *Nyctalus velutinus*  *alphacoronavirus SC-2013* | Chinese noctula  *Nyctalus velutinus* | Bats | Not established | Wu et al., 2018 |
|  |  | *Pedacovirus* | *Porcine epidemic diarrhea virus* | Sus scrofa domesticus  Domestic pig | Bats | Porcine epidemic diarrhea | Pensaert and de Bouck, 1978  Huang *et al*., 2013 |
|  |  |  | *Scotophilus bat coronavirus 512* | Lesser Asiatic yellow house bat  *Scotophilus kuhlii* | Bats | Not established | Tang et al., 2006 |
|  |  | *Rhinacovirus* | *Rhinolophus bat coronavirus HKU2* | Chinese horseshoe bat  *Rhinolophus* sinicus | Bats | Not established | Lau *et al*., 2007 |
|  |  | *Setracovirus* | *Human coronavirus NL63* | Human | Bats | bronchiolitis and conjunctivitis | van der Hoek *et al*., 2004 |
|  |  |  | *NL63-related bat coronavirus strain BtKYNL63-9b* | African trident bat  *Triaenops afer* | Bats | Not established | Tao *et al*., 2017 |
|  |  | *Tegacovirus* | *Alphacoronavirus 1^2^* | Cat (*Felis catus)*  Dog  *Canis familiaris*  Pig  *Sus Scrofa* | Bats |  | Carstens 2010 |
|  | *Betacoronavirus* | *Embecovirus* | *Betacoronavirus 1* | Human  Cattle (*Bos taurus)*  Giraffe (*Giraffa camelopardalis*)  Sable antelope (Hippotragus niger)  Sambar deer (Rusa unicolor)  Waterbuck (*Kobus ellipsiprymnus*)  White tailed deer (*Odocoileus virginianus*)  Dog *(Canis familiaris)*  Dromedary camel (*Camelus dromedarius*),  Horse (Equus caballus)  Pig (*Sus scrofa*)  Yak (*Bos grunniens*) | Bats | Enteric and respiratory disease in human and bovines  Respiratory disease in dogs  Porcine hemagglutinating encephalomyelitis  winter dysentery in sable antelopes and giraffes  anorexia, lethargy, and fever in horses | Woo *et al*., 2012 |
|  |  |  | *China Rattus coronavirus HKU24* | Norway rat  (Rattus norvegicus) | Rodents | Not established | Lau *et al.,* 2015 |
|  |  |  | *Human coronavirus HKU1* | Human | Bats | Associated with both upper and lower respiratory tract infections that are mostly self-limiting, pneumonia | Woo *et al*., 2005 |
|  |  |  | *Murine coronavirus* | Rodents | Rodents | Hepatitis and severe neurologic infection, resulting in paralysis and demyelination with high mortality, mainly in colonies of laboratory mice | Bailey *et al.,* 1949 |
|  |  | *Hibecovirus* | *Bat Hp-betacoronavirus Zhejiang2013* | Pratt's roundleaf bat  *Hipposideros pratti* | Bats | Not established | Wu *et al*., 2016 |
|  |  | *Merbecovirus* | *Hedgehog coronavirus 1* | Amur hedgehog  Erinaceus amurensis  European hedgehog  Erinaceus Europaeus | hedgehogs  Bats  recombination | Not established | Lau *et al*., 2019 |
|  |  |  | *Middle East respiratory syndrome-related coronavirus (MERS)* | Human  Domedary camel  *Camelus dromedarius*  Great evening bat (Ia io)  Asian particolored bats  Vespertilio superans  Chinese *Pipistrelle Bat*  *Hypsugo pulveratus*  Aloe serotine  Neoromicia cf. zuluensis | Bats  Dromedary  camels | Middle East Respiratory Syndrome (MERS) | WHO, 2017  Fan *et al*., 2019  Ithete *et a*l., 2013  Yusof *et al*., 2015  Chu *et al*., 2014 |
|  |  |  | *Pipistrellus bat coronavirus HKU5* | *Pipistrellus* bats | bats | Not established | Woo *et al*., 2006 |
|  |  |  | *Tylonycteris bat coronavirus HKU4* | *Tylonycteris* bats | bats | Not established | Woo *et al*., 2006 |
|  |  | *Nobecovirus* | *Rousettus bat coronavirus GCCDC1* | Leschenault's rousette fruit bat  *Rousettus leschenaultii* | bats | Not established | Huang *et al*., 2016 |
|  |  |  | *Rousettus bat coronavirus HKU9* | Leschenault's rousette fruit bat  *Rousettus leschenaultii* | bats | Not established | Woo *et al*., 2007 |
|  |  | *Sarbecovirus* | *Severe acute respiratory syndrome-related coronavirus (SARS)* | Human  Bats | Bats  masked palm civets  *Paguma larvata* | *Severe acute respiratory syndrome* | Wu *et al*., 2016 |
|  |  |  | *SARS-CoV-2 (SARS sister group)* | Human  Bats | Malayan pangolin  Bats | *Severe acute respiratory syndrome* | Zhou *et al*., 2020  Gorbalenya *et al*., 2020  Zhang *et al*., 2020 |
|  | *Gammacoronavirus* | *Cegacovirus* | *Beluga whale coronavirus SW1* | Beluga whale  *Delphinapterus leucas* | it is not yet clear whether beluga whales are the natural host, an amplifying host, or a dead-end host  Birds? | Illness characterized by generalized pulmonary disease and terminal acute liver failure | Wu *et al*., 2008 |
|  |  | *Igacovirus* | *Avian coronavirus* | Fowl  *Gallus gallus*  Pheasant  *Phasianus* spp*.*  nongalliform birds | birds | Infectious bronchitis | Boursnell *et al*., 1987 |
|  | *Deltacoronavirus* | *Andecovirus* | *Wigeon coronavirus HKU20* | widgeon  *Mareca penelope* | Birds | Not established | Woo *et al*., 2012 |
|  |  | *Buldecovirus* | *Bulbul coronavirus HKU11* | Chinese bulbul  *Pycnonotus sinensis*  Red*-*whiskered bulbul  *Pycnonotus jocosus* | Birds | Not established | Woo *et al*., 2009 |
|  |  |  | *Coronavirus HKU15* | Pig  *Sus Scrofa* | Birds? | Not established | Woo *et al*., 2012 |
|  |  |  | *Munia coronavirus HKU13* | White-rumped munia  *Lonchura striata*  Scaly-breasted munia  *Lonchura punctulata* | Birds | Not established | Woo *et al*., 2009 |
|  |  |  | *White-eye coronavirus HKU16* | warbling white-eye  *Zosterops japonicus* | Birds | Not established | Woo *et al*., 2012 |
|  |  | *Herdecovirus* | *Night heron coronavirus HKU19* | black-crowned night heron  *Nycticorax nycticorax* | Birds | Not established | Woo *et al*., 2012 |
|  |  | *Moordercovirus* | *Common moorhen coronavirus HKU21* | Common moorhen  *Gallinula chloropus* | Birds | Not established | Woo *et al*., 2012 |
|  |  |  |  |  |  |  |  |
